# The association between illness perception and family functioning with good death expectations in community-dwelling older adults with chronic diseases

**DOI:** 10.3389/fpubh.2026.2001715

**Published:** 2026-10-28

**Authors:** Yixuan Liang, Yingchun Wang

**Affiliations:** 1 School of Nursing, Jinzhou Medical University, Jinzhou, Liaoning, China; 2 First Affiliated Hospital of Jinzhou Medical University, Jinzhou, Liaoning, China

**Keywords:** chronic diseases, family functioning, good death expectations, illness perception, older adults

## Abstract

**Objective:**

To examine the association between good death expectations and family functioning in community-dwelling older adults with chronic diseases, and the association with illness perception.

**Methods:**

In this cross-sectional study (Nov 2025–Mar 2026) of 300 community-dwelling older adults (≥60 years, ≥1 chronic disease) from three districts of a city in China, validated tools measured key variables. Mediation analysis used PROCESS bootstrapping (5,000 samples) with sociodemographic adjustment.

**Results:**

Family functioning positively correlated with good death expectations (*r* = 0.561, *p* < 0.01) and negatively with illness perception (*r* = −0.482, *p* < 0.01). Illness perception negatively correlated with good death expectations (*r* = −0.506, *p* < 0.01). Illness perception showed a significant indirect effect in the relationship [indirect effect = 0.1317; 95% *CI* (0.0804,0.1912)], accounting for 26.12% of the total effect.

**Conclusion:**

Family functioning was associated with good death expectations and illness perception showed a significant indirect association. Future research is needed to determine whether interventions targeting these factors could improve end-of-life expectations.

## Introduction

Family functioning is defined as the systematic capacity of a family to address the needs of its members and foster overall development through emotional support and decision-making processes (1). Research indicates that effective family functioning is related to enhanced self-management abilities and reduced depressive symptoms among patients with chronic diseases, which may contribute to better quality of life (2, 3). Illness perception refers to the subjective cognitive evaluation and emotional responses that individuals exhibit when confronted with illness or health threats (4). A positive illness perception promotes active participation in health management, while a negative perception may lead to medical avoidance and a decline in quality of life (5). Furthermore, studies have confirmed that illness perception mediates the relationship between self-efficacy and health outcomes (6). Good death expectations encompass older adults ‘comprehensive aspirations regarding dignity preservation, symptom control, psychological support, and familial companionship during the end-of-life stage (7). According to Chen’s (8) conceptualization, these expectations include six dimensions: psychological and spiritual support, autonomy, respect, social support, healthcare system support, and physical comfort. A good death also implies that the dying individual can peacefully accept and appropriately manage socio-emotional and practical affairs before death, thereby ending life with tranquility and dignity (9, 10). The realization of these expectations is contingent not only upon the accessibility of medical resources but also on the family’s capacity to provide adequate support (11, 12).

While prior research has examined family functioning, illness perception, and good death expectations in isolation, the interrelationships among these three constructs remain insufficiently explored, particularly in the context of community-dwelling older adults with chronic diseases. Chronic illnesses often necessitate long-term management and may amplify perceptions of threat and uncertainty, potentially associated with how older adults reflect on death and dying.

A theoretical basis for these hypothesized relationships is provided by Leventhal’s Common-Sense Model (CSM) of self-regulation, which posits that individuals form cognitive and emotional representations of illness that guide their coping and health outcomes (13). According to CSM, these illness perceptions are shaped by external contextual inputs, including information and emotional feedback from the social environment. Drawing on the conceptual framework of family functioning articulated by Fang et al. (14), which synthesizes key dimensions of family dynamics in the Chinese context, we propose that cohesive family functioning reduces negative illness perceptions (e.g., perceptions of high threat or uncontrollability), which in turn enables older adults to engage in future-oriented thinking and develop more positive expectations regarding a good death. Thus, illness perception is theorized to serve as a cognitive mediator linking the distal family context to end-of-life expectations. We acknowledge that other variables—such as coping styles, social support, or spiritual well-being—may also play a role; however, illness perception was selected as the mediator due to its theoretical position as the most proximal cognitive pathway from contextual inputs to health expectations within the CSM framework.

In Chinese cultural contexts, where families play a pivotal role in providing both emotional and practical support, this theoretical pathway has particular clinical relevance. Therefore, this study aimed to (a) examine the correlations among family functioning, illness perception, and good death expectations in community-dwelling older adults with chronic diseases, and (b) test the mediating role of illness perception in the relationship between family functioning and good death expectations. It was hypothesized that better family functioning would be associated with higher good death expectations, and that this relationship would be partially mediated by lower levels of negative illness perception.

## Methods

### Study design and participants

This cross-sectional study was conducted between November 2025 and March 2026. Using convenience sampling, the study recruited 300 older adults (aged ≥60 years) with at least one chronic disease from three community health centers in a city in China (X City). Research assistants approached potential participants during routine health visits or community health promotion activities at the three centers. Eligible individuals were invited to participate voluntarily; no incentives were provided. The sample size was determined based on Beale et al. (15) recommended rule of thumb for observational studies, which requires a minimum of 5 to 10 participants per variable or dimension to ensure adequate statistical power. In this study, the total number of variables/dimensions included 15 sociodemographic items, 2 dimensions of family functioning (FACES), 3 dimensions of illness perception (BIPQ), and 6 dimensions of good death expectations (GDES), summing to 26 dimension variables. Applying the criterion proposed by Beale et al. (15), the required sample size ranged from 130 to 260 participants. Accounting for an anticipated 20% invalid response rate, the target sample size was adjusted to 162 to 324 participants. A total of 312 questionnaires were distributed, and 300 valid responses were obtained (valid response rate = 96.15%), which fell within the pre-specified target range. For the mediation analysis, a sample size of 300 exceeded the commonly recommended threshold of 200 for accurate estimation of indirect effects using bias-corrected bootstrap resampling (16, 17). The study was approved by the institutional ethics committee and was conducted in accordance with the principles of the Declaration of Helsinki. All participants provided informed consent prior to enrollment.

Inclusion criteria were: (a) age ≥60 years; (b) diagnosis of at least one chronic disease (e.g., hypertension, diabetes, coronary heart disease) confirmed by a medical institution; (c) conscious, without severe hearing or language impairments, able to communicate normally; and (d) voluntary participation. Exclusion criteria were: (a) severe organ failure or terminal illness; (b) significant cognitive impairment (Mini-Mental State Examination score <24); (c) severe mental illness or extreme emotional instability; and (d) major negative life events within the past 6 months (e.g., loss of spouse or major family upheaval).

### Diversity, equity, and inclusion considerations

The study was designed to be inclusive of diverse subgroups within the community-dwelling older adult population. The demographic questionnaire captured multiple dimensions of diversity, including gender (male/female), age (categorized into four groups: 60–69, 70–79, 80–89, ≥90), educational level (ranging from no schooling to high school/vocational school), living arrangements (urban vs. rural, living with family vs. alone vs. nursing home), marital status (married, divorced, widowed, unmarried), income source, primary caregiver (self, spouse/children, other relatives, paid caregiver/agency), and method of payment for medical expenses. These variables allowed the authors to examine whether the relationships among family functioning, illness perception, and good death expectations varied by social and economic background. Participants with lower educational levels or difficulty completing questionnaires independently received one-on-one assistance from trained investigators to ensure equitable participation. No participants were excluded on the basis of race or ethnicity because the study was conducted in a single geographic region with a predominantly Han Chinese population; however, the authors acknowledge that the findings may not generalize to other racial or ethnic groups.

## Measures

### Demographic questionnaire

A self-developed questionnaire based on existing literature assessed the following: gender, age, educational level, living arrangements, marital status, source of income, number and types of chronic diseases, frequency of hospitalizations in the past year, method of payment for medical expenses, self-assessed health status, primary caregiver, knowledge of chronic diseases, and level of understanding of advance medical directives.

### Family adaptability and cohesion evaluation scales

Developed by Olson and colleagues and adapted for the Chinese context by Fei et al. (18), the FACES measures family functioning. It is a core tool for assessing family functioning in older Chinese adults (19). The scale comprises two dimensions—Cohesion (16 items) and Adaptability (14 items)—for a total of 30 items. Each item is rated on a 5-point Likert scale (1 = almost never, 5 = almost always). Higher scores indicate more frequent endorsement of the described behavior. In this study, Cronbach’s α was 0.934.

### Brief Illness Perception Questionnaire

The BIPQ, optimized and simplified from the Illness Perception Questionnaire (20) and the Revised Illness Perception Questionnaire (21) by Broadbent, consists of 9 items across three dimensions: cognitive representations (Items 1–5), emotional representations (Items 6 and 8), and illness comprehensibility (Item 7). Item 9 (open-ended causal question) is not scored. Items 3, 4, and 7 are reverse-scored. Each item uses an 11-point Likert scale (0–10), with total scores ranging from 0 to 80; higher scores indicate greater perceived threat from the illness. Cronbach’s α in this study was 0.734.

### Good Death Expectation Scale

Developed by Chen (8), the GDES assesses older adults’ expectations of a good death across six dimensions: psychological and spiritual support, autonomy, respect, social support, healthcare system support, and physical comfort. The scale has 31 items, each rated on a 5-point Likert scale (1 = strongly disagree, 5 = strongly agree). Higher scores indicate stronger good death expectations. Cronbach’s α was 0.960.

### Statistical analysis

Data were analyzed using SPSS version 27.0 (IBM Corp., Armonk, NY, USA). Prior to conducting parametric analyses, we examined underlying statistical assumptions. Normality was assessed using Shapiro–Wilk tests and Q–Q plots, with skewness < |1.0| and kurtosis < |2.0| indicating acceptable normality. Linearity of bivariate relationships was confirmed through scatterplot inspection, with no evidence of curvilinear patterns. Homogeneity of variance was tested using Levene’s tests (all *p* > 0.05). Harman’s single-factor test indicated no significant common method bias (first factor explained 30.25% of variance, below 40% threshold (22)). Multicollinearity was also assessed, with tolerance values ranging from 0.668 to 0.909 and VIFs ranging from 1.007 to 1.590, all within acceptable limits. Categorical variables were summarized as frequencies and percentages, while continuous variables were expressed as means and standard deviations (M ± SD). Group comparisons were performed using independent-samples t-tests for two groups or one-way analysis of variance (ANOVA) for three or more groups. Effect sizes were calculated as partial η^2^ for ANOVA and Cohen’s d for independent-samples t-tests. Pearson correlation coefficients (r) were calculated to examine bivariate relationships among family functioning, illness perception, and good death expectations.

Mediation analysis was conducted using the PROCESS macro for SPSS (version 4.1 (17)), with illness perception serving as the mediator between family functioning (independent variable) and good death expectations (dependent variable). Bias-corrected bootstrap resampling (5,000 samples) was employed to estimate the indirect effect and its 95% confidence interval. Covariates included gender, age group, marital status, education level, living arrangements, number of chronic diseases, hospitalization frequency, and knowledge of chronic diseases. These sociodemographic and disease-related factors were selected based on their significant associations with good death expectations in univariate analyses (*p* < 0.05) and their theoretical relevance as potential confounders. Disease-related clinical variables (e.g., disease severity, functional status) were not included as covariates to avoid overcontrolling, as these may lie on the causal pathway between family functioning and illness perception. Statistical significance was set at α = 0.05 (two-tailed).

### Quality control

Trained researchers explained the study objectives and obtained informed consent before questionnaire administration. For participants with low educational levels or difficulty completing the questionnaire independently, investigators read each question aloud and recorded responses. Missing or inconsistent items were checked and supplemented on site. Of 312 distributed questionnaires, 12 were excluded due to incomplete or invalid responses, yielding 300 valid questionnaires (valid response rate = 96.15%).

## Results

### Participant characteristics and differences in good death expectations

In analyzing the results, the relationships among key variables were examined across demographic and social dimensions. All subgroup comparisons are presented in Table 1. Among the 300 participants, significant differences in good death expectations were observed by gender, age group, marital status, educational level, living arrangements, number of chronic diseases, hospitalization frequency in the past year, and level of knowledge about chronic diseases (all *p* < 0.05). No significant differences were found for primary caregiver type, self-rated health, medical payment method, source of income, or awareness of advance directives (all *p* > 0.05).

**Table 1:** Univariate analysis of good death expectations by participant characteristics (*N* = 300).

Characteristic	Category	*n* (%)	*M* (SD)	t/F	*p*	Effect size	Sig. *post hoc*
Gender	Male	159 (53.00)	127.06 (14.13)	*t* = 2.369^*^	< 0.05	*d* = 0.274	
	Female	141 (47.00)	122.80 (17.03)				
Age (Years)	60–69	137 (45.67)	126.77 (12.37)	*F* = 5.987^*^	< 0.05	η^2^ = 0.039	≥80 vs60–69, 70–79
	70–79	128 (42.67)	125.51 (14.79)				
	≥80	35 (11.67)	116.74 (25.43)				
Marital status	Married	238 (79.33)	127.40 (13.23)	*F* = 14.212^***^	< 0.001	η^2^ = 0.087	Married vs. Widowed, Unpartnered
	Unpartnered	15 (5.00)	118.47 (23.42)				
	Widowed	47 (15.67)	115.32 (19.70)				
Education level	Not in school	45 (15.00)	117.62 (23.85)	*F* = 5.511^***^	< 0.001	η^2^ = 0.053	No schooling vs. all others
	Elementary school	195 (65.00)	125.59 (13.48)				
	Middle school	37 (12.33)	126.73 (13.58)				
	High school/vocational school	23 (7.67)	132.39 (10.92)				
Living arrangements	Urban, with family	152 (50.67)	126.43 (12.70)	*F* = 5.932^***^	< 0.001	η^2^ = 0.057	Rural alone vs. all others
	Urban, alone	26 (8.67)	126.27 (13.90)				
	Rural, with family	101 (33.67)	125.49 (15.94)				
	Rural, alone	21 (7.00)	111.57 (27.11)				
Source of income	Pension	79 (26.33)	126.49 (16.85)	*F* = 1.588	0.192	η^2^ = 0.016	None
	Supported by children	185 (61.67)	125.38 (13.99)				
	Government	20 (6.67)	118.40 (21.93)				
	Labor income/Others	16 (5.33)	122.63 (18.22)				
Number of chronic diseases	1	86 (28.67)	128.56 (15.91)	*F* = 2.807^*^	< 0.05	η^2^ = 0.028	1 vs. 2, 3
	2	142 (47.33)	123.75 (14.71)				
	3	60 (20.00)	122.23 (17.37)				
	≥4	12 (4.00)	129.67 (12.12)				
Primary caregiver	Self	115 (38.33)	124.97 (13.15)	*F* = 0.656	0.580	η^2^ = 0.007	None
	Spouse/children	136 (45.33)	126.04 (15.73)				
	Other relatives	25 (8.33)	123.40 (13.24)				
	Nanny/caregiver/agency	24 (8.00)	121.63 (26.05)				
Hospitalizations (past year)	0 times	169 (56.33)	127.74 (13.23)	*F* = 6.177^*^	< 0.05	η^2^ = 0.040	0 vs. 1
	1 time	95 (31.67)	120.94 (19.94)				
	2 times or more times	36 (12.00)	123.36 (10.28)				
Medical payment method	Employee insurance	72 (24.00)	126.19 (20.60)	*F* = 1.525	0.208	η^2^ = 0.015	None
	Resident insurance/NCMS	187 (62.33)	125.57 (12.85)				
	Government-funded	18 (6.00)	123.28 (10.29)				
	Self-paid / others	23 (7.67)	118.75 (17.46)				
Awareness of advance directives	Unaware, no plan	164 (54.67)	124.61 (16.11)	*F* = 0.736	0.480	η^2^ = 0.005	None
	Aware, no plan	112 (37.33)	124.93 (15.00)				
	Aware, intend to make	24 (8.00)	128.75 (15.87)				
Self-rated health	Poor	41 (13.67)	122.44 (23.82)	*F* = 0.928	0.396	η^2^ = 0.006	None
	Fair	180 (60.00)	125.01 (13.64)				
	Good	79 (26.33)	126.54 (14.75)				
Knowledge of chronic diseases	Low	117 (39.00)	121.00 (18.14)	*F* = 8.088^***^	< 0.001	η^2^ = 0.052	Low vs. Lower-middle, Moderate or above
	Lower-middle	135 (45.00)	126.55 (12.38)				
	Moderate or above	48 (16.00)	130.77 (15.24)				

Good death expectation scores range from 31 to 155. Data are presented as M (SD) unless otherwise noted. F values are from one-way ANOVA; t value is from independent-samples t-test. Effect sizes are presented as η^2^ for ANOVA and Cohen‘s d for t-test. *Post-hoc* pairwise comparisons were performed using LSD. “None” indicates no significant pairwise comparisons; Percentages may not sum to 100 due to rounding. The symbols *, **, and *** indicate statistical significance at *p* < 0.05, *p* < 0.01, and *p* < 0.001, respectively.

### Descriptive statistics for key variables

Table 2 presents the total and subscale scores for family functioning, illness perception, and good death expectations.

**Table 2:** Total and subscale scores for family functioning, illness perception, and good death expectations (*N* = 300).

Project	Score range	*M* (SD)
Family functioning (total)	45–134	102.62 (15.35)
Intimacy	23–72	54.18 (8.81)
Adaptability	22–64	48.43 (7.25)
Illness perception (total)	1–47	22.41 (9.81)
Cognitive representation	1–31	14.44 (6.25)
Emotional representation	0–15	5.77 (3.89)
Illness comprehensibility	0–7	2.21 (1.83)
Good death expectations (total)	55–152	125.06 (15.67)
Psychological and emotional support	6–20	16.02 (2.22)
Autonomy	5–15	12.03 (1.78)
Respect	8–25	19.90 (2.86)
Social support	12–35	28.21 (3.87)
Healthcare system support	10–30	24.42 (3.31)
Physical comfort	9–30	24.50 (3.37)

### Bivariate correlations

As shown in Table 3, family functioning was positively correlated with good death expectations and negatively correlated with illness perception; illness perception was negatively correlated with good death expectations (all *p* < 0.01).

**Table 3:** Pearson correlations among family functioning, illness perception, and good death expectations (*N* = 300).

Variable	Family functioning	Illness perception	Good death expectations
Family functioning	1		
Illness perception	−0.482^**^	1	
Good death expectations	0.561^**^	−0.506^**^	1

*p* < 0.01 (two-tailed). Correlations are based on total scores. The symbol ** indicates statistical significance at *p* < 0.01.

### Mediation analysis

Bias-corrected bootstrap mediation analysis (5,000 resamples) indicated that illness perception showed a significant indirect association in the relationship between family functioning and good death expectations. The total effect of family functioning on good death expectations was 0.5043 (SE = 0.0529, *p* < 0.001). The direct effect was 0.3726 (SE = 0.0548, *p* < 0.001), and the indirect effect through illness perception was 0.1317 [*BootSE* = 0.0278, 95% CI (0.0804, 0.1912)], accounting for 26.12% of the total effect. All coefficients reported in the mediation model are unstandardized. The overall model explained 44.3% of the variance in good death expectations (*R^2^* = 0.4430, *F* = 22.9883, *p* < 0.001; see Table 4; Figure 1).

**Table 4:** Mediation analysis of illness perception in the relationship between family functioning and good death expectations.

Project	Effect size	SE	Boot 95% CI	Effect (%)
Total impact	0.5043	0.0529	0.4002–0.6084	100%
Direct impact	0.3726	0.0548	0.2648–0.4805	73.88
Indirect impact	0.1317	0.0278	0.0804–0.1912	26.12

Bootstrap resamples = 5,000; bias-corrected confidence intervals. All coefficients are unstandardized. Covariates included gender, age group, marital status, education level, living arrangements, number of chronic diseases, hospitalization frequency, and knowledge of chronic diseases.

**Figure 1:**
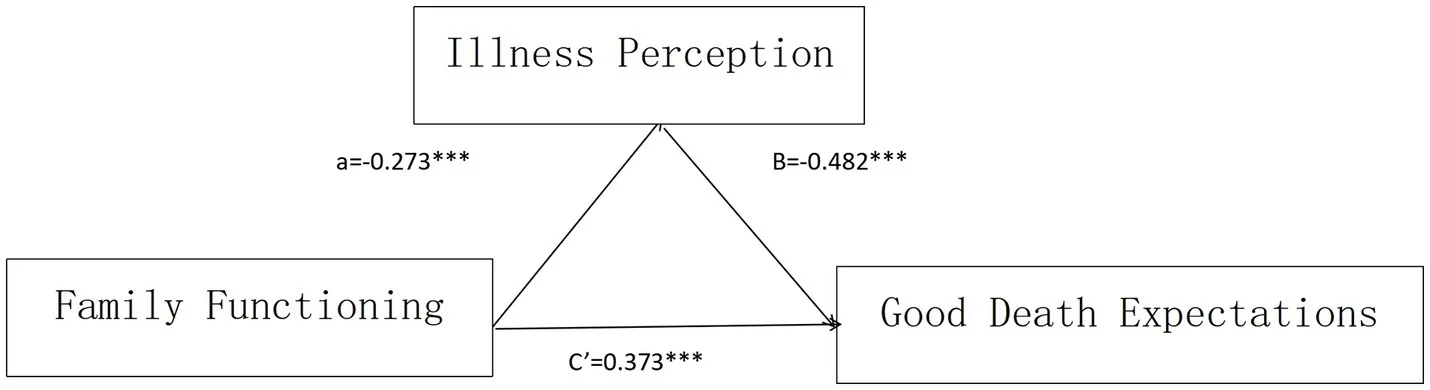
Exploratory model of the associations among illness perception, family functioning, and good death expectations. Exploratory model showing the associations among family functioning, illness perception, and good death expectations. The path from family functioning to illness perception is a = −0.273 (*p* < 0.001); the path from illness perception to good death expectations is b = −0.482 (*p* < 0.001). The direct association of family functioning with good death expectations, while adjusting for illness perception, is c′ = 0.373 (*p* < 0.001). All coefficients are unstandardized. All paths are statistically significant at the *p* < 0.001 level. The symbol *** indicates statistical significance at *p* < 0.001.

## Discussion

### Good death expectations among community-dwelling older adults with chronic diseases

The total score for good death expectations among community-dwelling older adults with chronic diseases was 125.06 (SD = 15.67). This descriptive result indicates that most older adults have some awareness and acceptance of a good death, but it does not imply a normative threshold. This result differs somewhat from the qualitative findings of Liu et al. (23); however, direct comparisons between quantitative and qualitative findings should be interpreted with caution given the different methodological approaches and sample characteristics. Among the six dimensions of good death expectations, the autonomy dimension showed the lowest mean score (*M* = 12.03, SD = 1.78). This dimension had the lowest mean score among the six dimensions, suggesting limited perceived control over end-of-life decisions, aligns with Zhang et al. (24), who reported a close association between weak autonomy and low acceptance of advance care planning among older adults. One possible interpretation is that older adults are currently navigating a transitional phase between traditional cultural values and modern quality-of-life concepts. Although some have begun to value expectations, public awareness of the good death concept remains insufficient, and cultural taboos around death continue to impede full acceptance. From a clinical perspective, our results suggest that assessing illness perception may be a relevant consideration for nursing practice. Specifically, the emotional representation dimension of the Brief Illness Perception Questionnaire (BIPQ) had a mean score of 5.77 (SD = 3.89) in our sample, and illness perception was significantly negatively correlated with good death expectations (*r* = −0.506, *p* < 0.001). Therefore, nursing assessments might reasonably include attention to patients’ emotional responses to their illness, although these clinical implications require confirmation in future intervention studies. Community nursing health education could integrate good death concepts into chronic disease management pathways, using family meetings and advance directive promotion. While not directly tested in this study, cognitive-behavioral approaches or motivational interviewing—which have shown efficacy in modifying illness perceptions in other chronic disease populations—warrant future investigation to determine whether they could be beneficial. Future research is needed to investigate whether interventions targeting illness perception could facilitate family discussions about end-of-life preferences. Furthermore, with respect to the specific dimensions of the Good Death Expectation Scale (GDES), the autonomy dimension scored lowest in our sample (*M* = 12.03, SD = 1.78, see Table 2), indicating that older adults may feel a lack of control over end-of-life decisions. This finding suggests that future studies should explore how specific dimensions of family functioning (e.g., communication and problem-solving) relate to specific dimensions of good death expectations.

### Illness perception among community-dwelling older adults with chronic diseases

The mean total score for illness perception was 22.41 (SD = 9.81), reflecting some degree of perceived threat from the illness. Specifically, the cognitive representation dimension had a mean score of 14.44 (SD = 6.25), and the emotional representation dimension had a mean score of 5.77 (SD = 3.89, see Table 2). This suggests that while older adults may have a moderate cognitive understanding of their illness, their emotional responses are also an important aspect to consider. Furthermore, the illness comprehensibility dimension had lowest mean score among the three BIPQ dimensions (*M* = 2.21, SD = 1.83), indicating limited understanding of their condition. These findings point to the potential value of exploring tailored interventions targeting both the cognitive and emotional aspects of illness perception among older adults with chronic diseases in future research. This may limit older adults’ understanding of disease severity, controllability, and consequences, thereby hindering effective self-management and health behaviors. This finding aligns with the research conducted by Ru et al. (25). A plausible explanation for this is that older adults often attribute age-related physiological declines to normal aging rather than to disease processes, which can lead them to overlook early symptoms and delay proactive management. Furthermore, limited access to health information and insufficient knowledge about disease prevention and treatment further impair their illness perception. These findings point to the potential value for future practice and research to explore health education strategies that distinguish normal aging from disease indicators. For example, future studies could examine the effectiveness of utilizing symptom comparison charts to improve older adults’ ability to identify nonspecific health risks, as well as community-based approaches such as health lectures and digital media to teach basic disease monitoring. However, it is important to note that these suggestions are hypothesis-generating and require further investigation to establish their potential clinical utility.

### Correlations among family functioning, illness perception, and good death expectations

Family functioning was significantly positively correlated with good death expectations (*r* = 0.561, *p* < 0.001), consistent with Wang et al. (26) based on the McMaster Model. The McMaster Model emphasizes that family functioning is a multidimensional construct encompassing problem-solving, communication, and affective responsiveness. Our findings support the applicability of this model in the context of end-of-life expectations, suggesting that a harmonious family environment, characterized by effective communication and mutual support, is associated with more positive expectations for a good death among older adults. The family functioning total score was 102.62 (SD = 15.35), with the intimacy dimension scoring 54.18 (SD = 8.81) and the adaptability dimension scoring 48.43 (SD = 7.25, see Table 2). These results suggest that families with higher cohesion and adaptability may provide a stronger supportive environment for older adults, which is consistent with the core assumptions of the McMaster Model. This suggests that family dimensions such as problem-solving, emotional responsiveness, and behavioral control constitute a psychological resource pool that supports older adults in coping with major life events. A harmonious family atmosphere and adequate support likely provide emotional comfort and security, which may help older adults face end-of-life with greater composure and enhance their acceptance of a good death. The results also confirmed that family functioning was negatively correlated with illness perception, and illness perception was negatively correlated with good death expectations (both *p* < 0.001). These findings align with Zhang et al. (27) and Karademas (28), who argued that good family functioning reduces patients’ perception of illness threat. Conversely, when patients develop high-threat, uncontrollable illness schemas, they may activate negative coping strategies (e.g., avoidance, resignation), which diminish meaning in life and acceptance of death, thereby limiting the psychological resources needed for a good death.

### Illness perception and the association between family functioning and good death expectations

The exploratory analysis showed that illness perception had a significant indirect association in the relationship between family functioning and good death expectations [indirect effect = 0.1317; 95% CI (0.0804,0.1912)], accounting for 26.12% of the total effect. This finding is consistent with the interpretation that family functioning may be related to good death expectations both directly and indirectly through illness perception. Specifically, better family functioning was associated with older adults taking an active interest in their health and seeking disease-related information. Furthermore, illness perception was associated with how older adults plan their health and daily lives, which may be related to more positive end-of-life expectations. The findings highlight the potential value for healthcare providers to consider the critical role of family functioning. Through home visits and family meetings, they can guide family members to optimize emotional interaction patterns and increase communication about end-of-life topics, thereby potentially enhancing psychological security. Regarding the pathway through illness perception, cognitive-behavioral interventions may help older patients acknowledge physiological limitations imposed by illness and transform illness-related reflections into motivation for life planning (e.g., participating in advance care planning discussions). Incorporating good death education into community chronic disease management, utilizing strategies like motivational interviewing and the promotion of advance directives, may help older adults articulate their medical preferences with the backing of their families. If supported by future research, this pathway—family support → cognitive management → end-of-life expectations—could inform the development of structured interventions to improve end-of-life quality of life.

### Limitations and future directions

Several limitations should be noted. First, given the cross-sectional design, the mediation analysis should be considered exploratory and cannot establish causality. Second, convenience sampling from three communities in one city limits generalizability. Third, self-report measures may be subject to social desirability and common method bias, potentially inflating observed correlations; future studies could incorporate objective assessments. Fourth, the absence of qualitative data limits deeper understanding of participants’ lived experiences. Fifth, Chinese cultural taboos surrounding death and Confucian filial piety may have shaped responses, restricting cross-cultural transferability. Sixth, clinical variables such as disease severity, functional status, and depression were not controlled and may confound results. Finally, the correlational design does not support strong practice recommendations; findings are hypothesis-generating.

In addition to these limitations, several alternative explanations warrant consideration. While our findings are consistent with the hypothesized indirect pathway, reverse directionality cannot be ruled out: older adults who hold more positive expectations regarding a good death may be more psychologically resilient, which could lead them to perceive their illness as less threatening and their family interactions as more supportive, rather than family functioning driving these perceptions. Second, unmeasured third variables, such as dispositional optimism, religious or spiritual beliefs, or prior experiences with end-of-life care in the family, may underlie all three constructs and account for part of the observed associations. Third, the partial indirect effect (26.12% of total effect) and the overall model *R*^2^ of 0.4430 indicate that approximately 55.7% of the variance in good death expectations remains unexplained. This suggests that additional pathways—such as psychological security, practical decision-making support, or direct modeling of death attitudes within families—may operate alongside or instead of illness perception. Despite these limitations, this study provides initial quantitative evidence for the potential role of illness perception in the family functioning–good death expectations relationship, and offers a foundation for future longitudinal and intervention research to build upon.

Future research should employ longitudinal or experimental designs, diverse samples, mixed methods, and intervention studies targeting family functioning and illness perception to improve end-of-life expectations.

## Conclusion

This cross-sectional study found that family functioning was positively associated with good death expectations among community-dwelling older adults with chronic diseases, and illness perception demonstrated a significant indirect association. Nurses caring for this population may consider attending to both family dynamics and older adults’ cognitive and emotional representations of their illnesses. Given the correlational design, findings are hypothesis-generating only. Longitudinal and intervention studies are needed to formally test these potential pathways and establish temporal precedence. Until then, pragmatic efforts may focus on facilitating open family dialogs about illness experiences and end-of-life preferences within comprehensive chronic disease management.

## Data Availability

The raw data supporting the conclusions of this article will be made available by the authors, without undue reservation.
